# Evaluation of Arch Dimensions and Bolton Ratio in Manual and Artificial Intelligence Tooth Segmentation

**DOI:** 10.1155/tswj/7598703

**Published:** 2026-05-15

**Authors:** Sara M. Al-Mashhadany, Dheaa Al-Groosh, Ammar Sh. Al-Ubaydi

**Affiliations:** ^1^ Orthodontic Department, College of Dentistry, University of Baghdad, Baghdad, Iraq, uobaghdad.edu.iq; ^2^ Orthodontic Department, Ministry of Health, Baghdad, Iraq, behdasht.gov.ir

**Keywords:** arch dimension, artificial intelligence, Bolton ratio, segmentation

## Abstract

**Objective:**

A 3D tooth model segmented with the use of deep learning (DL) method program (CephX) as well as MIMICS software (manual segmentation) will be evaluated for reliability in the presented work. In addition, the segmented model was compared with the intraoral scan (IOS)–generated 3D tooth model in terms of the Bolton ratio and arch dimensions.

**Methods and Materials:**

A total of 30 patients attending the College of Dentistry/University of Baghdad with records of IOSs and CBCT scans were included. CBCT has been transformed into a 3D digital tooth model segmented with the use of MIMICS software and an AI‐based program (CephX), and the Bolton ratio and arch dimensions (length and width) were measured utilizing Geomagic Control X software. Statistical analyses, including the mean and standard deviation, were performed, and a paired *t*‐test was used to assess the systematic bias between the three methods. Bland–Altman plots and intraclass correlation (ICC) analysis were used to assess the agreement between the three methods.

**Results:**

The means of CephX, MIMICS, and IOS of all analyses were mostly similar, and the difference between them was greatest in the anterior Bolton ratio of IOS and CephX images. The systematic bias demonstrated no significant difference (*p* value > 0.05) between CephX and MIMICS, but CephX versus IOS and MIMICS versus IOS demonstrated significant differences (*p* value < 0.05) in several measurements, such as Bolton ratios and arch length. Agreement using ICC revealed good to excellent reliability overall, but moderate agreement in overall Bolton between MIMICS‐IOS and between CephX‐IOS, anterior Bolton and interpremolar distance between CephX‐MIMICS, and poor agreement in anterior Bolton between MIMICS‐IOS and between CephX‐IOS was found. Bland–Altman plots showed that CephX and MIMICS were consistent, implying minimal systematic bias.

**Conclusions:**

Digital and AI‐driven tooth segmentation (MIMICS and CephX), and IOS methods generally provide consistent and reliable measurements in terms of the Bolton ratio and arch dimensions. However, caution is advised when interpreting certain Bolton ratio values because discrepancies and lower agreement may occur.

## 1. Introduction

Accurate orthodontic diagnosis and treatment planning are made possible by orthodontic study models, clinical examination of the patient, and radiological evaluation [1, 2]. In this context, particularly Bolton analysis and arch measurements are among the different orthodontic study model analyses that have been suggested to identify abnormalities in teeth and arch sizes [2], which may impact the final occlusal and aesthetic outcomes [3, 4]. The use of digital models has several potential benefits, including the ability to create numerous diagnostic setups, ease of use on computer screens, and the potential to lower long‐term costs [5, 6].

The correct evaluation of the dimensions of the dental arch and the size of teeth is the key to the success of orthodontic treatment; the Bolton analysis is used as the standard for measuring tooth‐size discrepancies. Traditionally, the method was carried out manually, comparing the mesiodistal widths of both mandibular and maxillary teeth using two ratios: the overall ratio, which is an average of 12 teeth per arch, and the anterior ratio, which is the average of six anterior teeth [7]. Such measurements assist clinicians in identifying an imbalance and facilitate interproximal reduction (IPR), extractions, or restoration planning, as well as attaining optimal occlusion and alignment [8]. With the integration of artificial intelligence (AI) and digital modeling, Bolton analysis has become highly efficient and accurate, improving clinical decision‐making and simplifying orthodontic workflows by importing intraoral scanner (IOS) [9].

AI has made it possible to segment and identify complex imaging data in dental radiology. In just a few minutes, it can automatically transform DICOM files from CBCT scans into segmented 3D images in STL format [10], and the automatic segmentation method demonstrated good reliability and accuracy in three‐dimensional aspects [11]. In order to help clinicians with orthodontic treatment planning, a variety of digital model software tools were developed recently [5, 11–13]; accordingly, further studies are required to assess the reliability of these tools. So, the purpose of the presented work is to evaluate the segmented 3D tooth model′s reliability with the use of an AI‐assisted program (CephX), manually segmented utilizing MIMICS software, and after that compare it with the IOS′s 3D tooth model, which is regarded as standard due to high accuracy and reproducibility for tooth and arch dimensions according to previous researches [14–16].

## 2. Materials and Methods

### 2.1. Sample

This retrospective study was approved by the Research and Ethics Board Committee of the College of Dentistry, University of Baghdad (No. 624422/2021) with a written consent form for all participants. A sample size calculation was performed using G ^∗^Power (Version 3.1.9.4, Win) [17] to determine the minimum number of participants required to detect a statistically significant difference between manual, AI‐assisted, and IOS groups. The priori analysis was done, assuming a medium effect size (Cohen^′^s dz = 0.45), a statistical power (1–*β*) of 0.85, and (*α*) level of 0.05 [9]. Based on these parameters, the minimum required sample size was calculated to be 30 patients.

Digital records of 30 patients enrolled at the University of Baghdad′s College of Dentistry who met the following inclusion criteria: Class I malocclusion, age between 15 and 30 years with mild‐to‐moderate crowding (irregularity index < 6 mm), and a complete set of permanent teeth. Patients with large fillings, significant prosthodontic or restorative therapy, malformed tooth shape, severe root resorption, or dilacerated roots were excluded. The participants′ records included CBCT and IOS images.

### 2.2. Methodology

**Figure 1:**
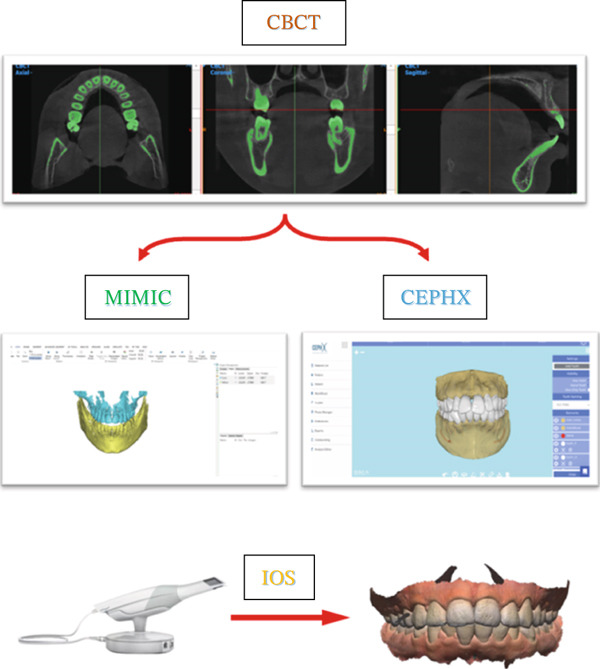
Steps of sample preparation.

**Figure 2:**
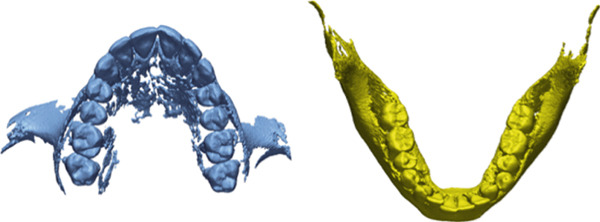
Segmentation of the upper and lower arches using MIMICS software.

**Figure 3:**
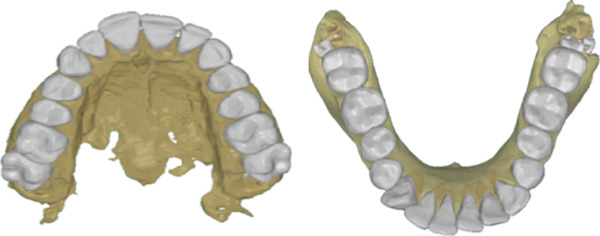
Segmentation of the upper and lower arches using CephX web viewer.

**Figure 4:**
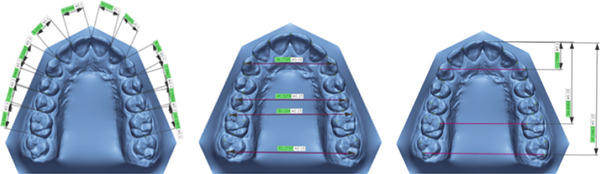
Dental and arch measurements using Geomagic Control X software.

CBCT scans for all patients were taken using a SOREDEX (Tuusula, Uusimaa, Finland) with a voxel size of 0.39 mm and a field of vision of 5 mA, 80 kV, and 179 × 200 mm, along with the IOS for the same patient via a 3D optical laser scanner (3Shape TRIOS 3, Copenhagen, Denmark) [18]. CRANEX software (Version 2.1.0.30) was used to export the CBCT in DICOM format and saved on recordable media (Figure 1). The DICOM files were sent to MIMICS software (v 21.0.0 Materialize HQ, Leuven, Belgium), and the CBCT had predefined thresholds that were set to correspond to the tooth or bone density as follows: tooth, 1200–3071 segments and bone, 226–3071 segments. The threshold level was set to most clearly show the tooth anatomy with minimal interference from the surrounding bone and adjacent structures. On each CBCT slice, manual refinement was performed through a 2D slice‐by‐slice procedure to enhance accuracy by correcting for overcontoured and undercontoured voxels in the tooth volume. The jaws and teeth were manually segmented and converted into STL format (Figure 2), The same DICOM files were sent to the CephX web viewer (Orca Dental Al, Las Vegas, Nevada) uploaded, and then jaws and teeth were automatically segmented in STL format after a few minutes (Figure 3). STL files of both MIMICS and CephX were imported into Geomagic Control XTM software (3D Systems Inc., Rock Hill, United States) to measure the following parameters for IOS, manually segmented MIMICS, and automatically segmented CephX (Figure 4):

1. Bolton ratios [4, 19]: the mesiodistal width of every tooth anterior to the second molars, measured at the mesial and distal contact points parallel to the occlusal surfaces. The following is the Bolton analysis calculation.

Overall Bolton ratio= sum of mandibular 12 teethsum of maxillary 12 teeth×100Anterior Bolton ratio= sum of mandibular 6 teethsum of maxillary 6 teeth×100

2. Dental arch dimensions of the maxilla were as follows:
  i. Dental arch width, which includes intercanine distance (the horizontal distance between the upper canines′ cusp tips) [4, 20], interpremolar distance (the horizontal distance between the buccal cusp tips of the second premolars) [3], inter‐first molar distance (the horizontal distance between the mesiobuccal cusp tips of the first molars) [4, 20], and inter‐second molar distance (the horizontal distance between the distobuccal cusp tips of the second molars) [4].
  ii. Dental arch length [4], which includes anterior arch length (from the incisal point to a line between the cusp tips of canines), molar vertical distance (from the incisal point to a line connecting mesiolingual cusp tips of the first molars), and total arch length (from the incisal point to a line connecting distobuccal cusp tips of the second molars).

## 3. Statistical Analysis

Statistical Package for Social Sciences (SPSS) v. 26.0 (IBM SPSS, Armonk, New York) was used for data management. Descriptive statistics (maximum, minimum, mean, and standard deviation) were computed for the three groups. The Shapiro–Wilk test for the normality of the data distribution was employed, and a paired *t*‐test was used to evaluate the systematic bias among the three groups. The agreement between the measurement of segmentation produced by CephX web viewer, MIMICS software, and IOS was examined using intraclass correlation (ICC) analysis, and Bland–Altman plot for the agreement between CephX and MIMICS groups. The level of agreement was described as follows: ICC < 0.50 (poor agreement), ICC 0.50–0.75 (moderate agreement), ICC 0.75–0.90 (good agreement), and ICC > 0.90 (excellent agreement) [21]. A 95% confidence interval was determined, and a probability value of 0.05 was established.

## 4. Results

Ninety records of 30 patients were divided into three groups based on segmentation type (30 CBCT images segmented using the AI‐based CephX program, 30 CBCT images manually segmented using the MIMICS program, and 30 IOS images, which were regarded as the standard).

### 4.1. Interrater Reliability

Five sets of models have been measured daily by two skilled orthodontists working independently utilizing Geomagic Control XTM software to prevent eye fatigue as well as reduce the possibility of subjective error. Ten models were randomly chosen and remeasured after a month to evaluate interrater repeatability. ICC revealed an excellent interrater reliability (0.96–0.99) and interrater repeatability (0.94–0.96).

### 4.2. Normality

The Shapiro–Wilk normality tests showed no significant deviation from normal distribution, with *p* values higher than 0.05 in all groups.

### 4.3. Descriptive Statistics

Tables 1 and 2 show that the descriptive statistics indicated the absence of missing data, and the mean for each method was close to each other, with the largest mean difference being in the anterior Bolton ratio between IOS and CephX (−2.29).

**Table 1:** Descriptive statistics for the measurements of MIMICS, CephX, and IOS.

Variable	Software	*N*	Mean	Standard deviation	Minimum	Maximum
Overall Bolton	MIMICS	30	91.52	1.46	89.24	94.48
	CephX	30	91.29	1.73	89.05	95.07
	IOS	30	92.69	1.45	90.17	95.23

Anterior Bolton	MIMICS	30	79.79	2.28	74.79	82.23
	CephX	30	78.20	2.38	74.54	82.96
	IOS	30	80.49	2.31	77.98	84.77

Intercanine distance	MIMICS	30	35.18	1.13	32.82	36.81
	CephX	30	34.72	1.68	30.57	36.44
	IOS	30	34.93	1.75	31.52	37.32

Interpremolar distance	MIMICS	30	46.02	2.26	42.58	49.67
	CephX	30	45.67	2.47	41.71	49.20
	IOS	30	45.79	2.36	41.36	48.45

Inter‐first molar distance	MIMICS	30	51.12	2.63	46.36	55.42
	CephX	30	50.76	2.69	46.39	54.84
	IOS	30	50.82	2.54	46.33	55.22

Inter‐second molar distance	MIMICS	30	59.94	4.97	51.71	65.40
	CephX	30	59.07	4.44	51.66	64.29
	IOS	30	59.31	4.57	51.95	64.40

Anterior arch length	MIMICS	30	7.90	1.54	5.25	10.37
	CephX	30	8.61	1.18	6.53	9.91
	IOS	30	7.88	1.49	5.12	10.12

Molar vertical distance	MIMICS	30	32.06	3.09	26.40	36.22
	CephX	30	32.48	2.81	28.33	37.60
	IOS	30	31.68	2.95	26.40	35.85

Total arch length	MIMICS	30	44.22	3.19	38.86	48.65
	CephX	30	44.43	3.02	39.37	48.41
	IOS	30	43.51	3.69	35.68	48.11

**Table 2:** Paired *t*‐test for the assessment of systematic bias between the MIMICS, CephX, and IOS.

Paired variables		Mean difference	SD	SE	95% confidence interval of the difference lower upper		*t*‐test	*p* value
MIMICS‐CephX	**Overall Bolton**	0.23	1.48	0.47	−0.83	1.29	0.49	0.64
	**Anterior Bolton**	1.59	2.32	0.73	−0.07	3.24	2.16	0.06
	**Intercanine distance**	0.46	1.02	0.32	−0.27	1.19	1.43	0.19
	**Interpremolar distance**	0.34	2.54	0.80	−1.47	2.16	0.43	0.68
	**Inter-first molar distance**	0.36	0.61	0.19	−0.08	0.80	1.87	0.09
	**Inter-second molar distance**	0.86	1.72	0.54	−0.37	2.09	1.59	0.15
	**Anterior arch length**	−0.71	1.03	0.33	−1.45	0.03	−2.17	0.06
	**Molar vertical distance**	−0.42	1.44	0.46	−1.46	0.61	−0.93	0.38
	**Total arch length**	−0.21	0.41	0.13	−0.50	0.08	−1.62	0.14

MIMICS‐IOS	**Overall Bolton**	−1.17	1.23	0.39	−2.04	−0.29	−3.01	**0.01**
	**Anterior Bolton**	−0.70	2.81	0.89	−2.71	1.31	−0.79	0.45
	**Intercanine distance**	0.25	0.80	0.25	−0.32	0.83	1.00	0.34
	**Interpremolar distance**	0.23	1.56	0.49	−0.88	1.34	0.47	0.65
	**Inter-first molar distance**	0.30	0.57	0.18	−0.10	0.71	1.69	0.12
	**Inter-second molar distance**	0.63	1.43	0.45	−0.39	1.65	1.39	0.20
	**Anterior arch length**	0.02	0.29	0.09	−0.19	0.23	0.24	0.82
	**Molar vertical distance**	0.38	0.38	0.12	0.11	0.66	3.18	**0.01**
	**Total arch length**	0.70	0.95	0.30	0.03	1.38	2.35	**0.04**

CephX‐IOS	**Overall Bolton**	−1.40	1.33	0.42	−2.35	−0.44	−3.32	**0.01**
	**Anterior Bolton**	−2.29	3.01	0.95	−4.44	−0.13	−2.40	**0.04**
	**Intercanine distance**	−0.21	0.82	0.26	−0.79	0.38	−0.80	0.44
	**Interpremolar distance**	−0.12	1.65	0.52	−1.30	1.07	−0.22	0.83
	**Inter-first molar distance**	−0.06	0.67	0.21	−0.54	0.42	−0.28	0.78
	**Inter-second molar distance**	−0.23	0.71	0.23	−0.74	0.27	−1.04	0.32
	**Anterior arch length**	0.73	1.06	0.33	−0.03	1.49	2.18	0.06
	**Molar vertical distance**	0.81	1.27	0.40	−0.10	1.71	2.02	0.07
	**Total arch length**	0.91	1.09	0.35	0.13	1.69	2.65	**0.03**

*Note:* Bolded data indicate p *p* ≥ 0.05.

### 4.4. Systematic Bias

Table 2 shows that the paired *t*‐test revealed no statistically significant differences in any of the measurements between CephX and MIMICS. The comparison between CephX and IOS shows no significant difference in all measurements apart from the overall Bolton ratio, molar vertical distance, and total arch length (*p* ≥ 0.05), whereas the overall Bolton, anterior Bolton, and total arch length demonstrated a significant difference between MIMICS and IOS (*p* < 0.05) and no difference for the other measurements.

### 4.5. Agreement

Table 3 shows that the ICC test demonstrated good to excellent agreement for all measurements between CephX, MIMICS, and IOS, with values ranging from 0.75 to 0.99, with the exception of moderate agreement in overall Bolton between MIMICS and IOS, between CephX and IOS, anterior Bolton between CephX and MIMICS, and interpremolar distance between CephX and MIMICS, whereas poor agreement was found in anterior Bolton between MIMICS and IOS and between CephX and IOS.

**Table 3:** Intraclass correlation coefficient for the measurements between MIMICS, CephX, and IOS.

Variables	MIMICS‐CephX	MIMICS‐IOS	CephX‐IOS
Overall Bolton	0.75	0.67	0.67
Anterior Bolton	0.61	0.45	0.27
Intercanine distance	0.84	0.92	0.94
Interpremolar distance	0.63	0.88	0.88
Inter‐first molar distance	0.98	0.98	0.98
Inter‐second molar distance	0.96	0.97	0.99
Anterior arch length	0.79	0.99	0.76
Molar vertical distance	0.93	0.99	0.91
Total arch length	0.99	0.97	0.95

**Figure 5:**
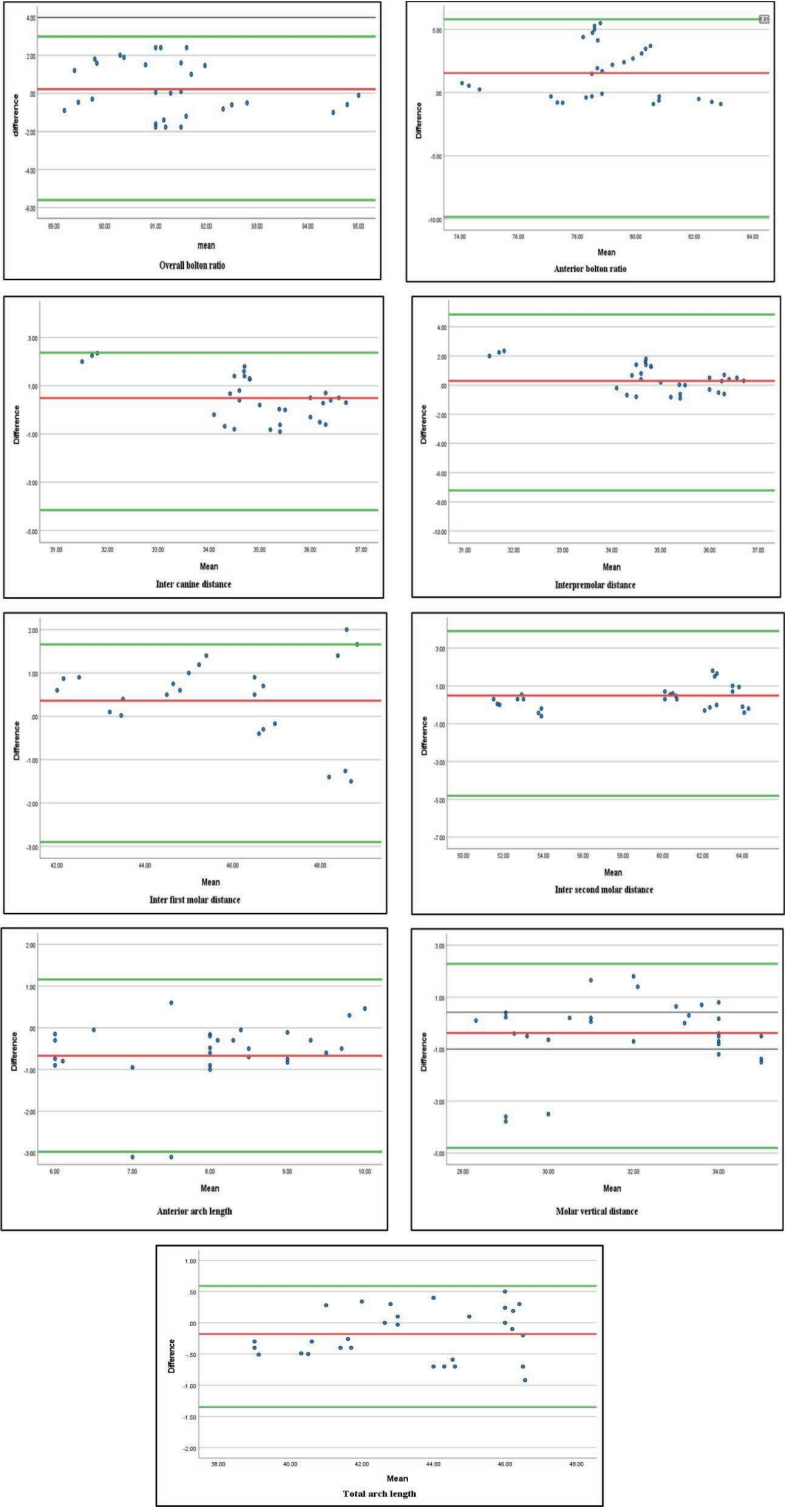
Bland–Altman plot of means and differences of all measurements for both MIMICS and CephX software.

The Bland–Altman plot demonstrated reasonable consistency between CephX and MIMICS for all measurements with 95% limits of agreement, and most points were close to the mean difference line and within the limit of agreement, indicating minimal systematic bias in anterior arch length and inter first molar distance (Figure 5).

## 5. Discussion

Automatic teeth segmentation is a recent technology in orthodontics that allows an accurate 3D representation of the oral cavity, improves diagnostic accuracy, enhances patient communication through visual aids, and streamlines clinical workflows. An advanced segmentation algorithm (between thresholding and region growing, to deep learning) is then used to separate individual teeth, with the disadvantage of the inability to distinguish closely positioned teeth, variability of teeth morphology, and metal artifacts [22].

The two platforms now allow the import of CBCT data, and whereas MIMICS manually segments CBCT, CephX currently offers AI‐assisted tooth segmentation straight from the CBCT scans and does not appear to calculate arch dimension(arch width and length) and Bolton ratio automatically from segmented CBCT; other programs (e.g., Ortho3Shape and OrthoKit) can offer only digital model analysis but not for segmented CBCT, different aspects have been studied by several researches on individually segmented tooth although it is essential to measure these parameter in the segmented teeth within the whole arch such as arch dimensions and Bolton analysis, which will be discussed in the current study in two sections.

For the Bolton ratio, this study demonstrates that the mean of the three methods for both overall and anterior Bolton ratio was close to each other, being larger in IOS than MIMICS and lowest in CephX, but these differences were generally not clinically significant; agreement was generally good between MIMICS and CephX and moderate between both MIMICS and CephX with IOS for overall Bolton, and minimal agreement was in anterior Bolton between CephX and IOS, with a significant mean difference between them of more than 2, which is clinically not acceptable; Crosby and Alexander [2] explained that a difference within 1–1.5 in Bolton ratio was acceptable, while exceeding 2 is considered clinically significant, as they may reflect true tooth‐size disharmony requiring treatment modification.

Bolton analysis measured using emodel software could be as precise as and much faster than the conventional method of digital calipers and plaster models according to Mullen et al. [6]. Teixeira et al. [23] found that ClinCheck and Dolphin software provide reliable anterior Bolton measurements and a moderate reliability for overall Bolton ratios, in contrast with the study by Shailendran et al. [24] who found that the ClinCheck Pro software generally underestimates tooth widths compared with 3D optical profilometry, leading to inaccurate and clinically unacceptable Bolton ratios. These findings may be due to “shape assumptions” in the interproximal areas.

Pasini et al. [25] demonstrated that digital technology, including extraoral scanners, facilitates the accurate measurement of the Bolton ratio, and found digital models to be a reliable alternative to plaster models for preserving and retrieving data. In comparison with caliper measurements, Kumar et al. [26] found that OrthoAnalyzer software yielded greater values for tooth width and Bolton′s ratio, indicating that plaster models remain the most accurate method for assessing tooth widths and Bolton′s ratios.

According to Bor et al. [9], there was limited agreement between the diagnostic accuracy of a particular AI program (Titan Dental Design and SoftSmile programs) and that of a group of experienced orthodontists for overall and anterior Bolton.

For the arch dimension, the present study showed that the mean values of the three methods for both width and length were similar, with no significant difference between them, and demonstrated good to excellent agreement among the three methods.

Lang et al. [27] studied orthodontic model analysis using plaster casts and virtual models evaluated with OnyxCeph3TM and found that partially automated digital cast analysis is a precise, incredibly effective, and also time‐saving substitute for conventional manual cast analysis.

All characteristics, such as arch length, overjet, and tooth width, showed no statistically significant differences between digital and plaster models, according to Quimby et al. [28]. Comparably, Yu et al. [10] compared manual and automatic digital model analyses utilizing IOS models, and the automatic technique showed reproducible analysis in a significantly shorter time, and the measurements of Bolton as well as arch dimension differed significantly from the manual approach.

Sinard et al. [29] study different software used for automatic teeth segmentation from CBCT such as ReLU, Diagnocat, CephX, and DentalSegmentator, which showed good results in terms of segmentation accuracy.

The studies collectively show a revolutionary shift in orthodontics toward digital and automated segmentation, highlighting the significance of reliability and accuracy in the assessment of arch dimensions and Bolton ratios. The incorporation of AI into such procedures will improve clinical procedures in dental care and diagnostic capabilities [30, 31], and the manual segmentation techniques may result in a bias. Interobserver variability could be the source of such biases, which could be especially difficult when working with large datasets or complex anatomical structures [32].

The primary drawback of this study is that the reliability was evaluated on a small sample size (*n* = 30), which might have restricted the power of our work. Future research with a larger sample size is recommended to improve the generalizability and robustness of the results. Additionally, it would be better to evaluate the segmentation time and different types of malocclusions to provide a more comprehensive evaluation.

## 6. Conclusions

Digital and AI‐driven tooth segmentation (MIMICS and CephX), and IOS methods generally provide consistent and reliable measurements for the Bolton ratio and arch dimension analysis in orthodontics. These methods are largely interchangeable for most dental measurements, showing high agreement and minimal bias. However, clinicians should be cautious with certain Bolton ratio measurements, where moderate‐to‐poor agreement and significant differences may occur.

**Nomenclature**
DICOM: Digital Imaging and Communications in Medicine
STL: stereolithography
IOS: intraoral scanner
CBCT: cone‐beam computed tomography

## Author Contributions

Conceptualization, methodology, data curation, and writing of the original draft: S.M.A‐M.; validation, formal analysis, investigation, and resources: A.S.A‐U.; writing, review, editing, visualization, and supervision: D.A‐G.

## Funding

No funding was received for this manuscript.

## Conflicts of Interest

The authors declare no conflicts of interest.

## Data Availability

The corresponding author can provide the data to support the findings of this study upon request.
